# Supplementary material for: Morphology of the Female Reproductive System of the Soybean Thrips, Neohydatothrips variabilis (Beach, 1896) (Thysanoptera: Thripidae)
Source: Insects. 2022 Jun 23;13(7):566. doi: 10.3390/insects13070566 (PMC9317315; doi:10.3390/insects13070566)
Supplement: Supplementary file 1 [file insects-13-00566-s001.zip › insects-1777683-supplementary.pdf]

Supplementary Materials:

Table S1. Terminologies used in the paper.

Abbreviation	English Terminology	German Translation of the terminology	Terminology first proposed by
ADR	Appendage gland	Anhangsdrüse	Bode [29]
V1	1st gonapophysis or 1st valvula		Snodgrass [35],[36]
V2	2nd gonapophysis/ 2nd valvula		Snodgrass [35],[36]
1.Vf	1st Valvifer		Snodgrass [35],[36]
2. Vf	2nd Valvifer		Snodgrass [35],[36]
AP	Apodeme		Snodgrass [35],[36]
FOC	follicle cells		Snodgrass [35],[36]
ET	Epithelial layer		Snodgrass [35],[36]
NC	Nutrition cells		Snodgrass [35],[36]
Yo	Yolk		Snodgrass [35],[36]
MO	Matured oocytes		Snodgrass [35],[36]
IO	Immature oocytes		Snodgrass [35],[36]
GC	gland cell	einzelnen Drüsenzellen	Bode [29]
WC	Wall cell	Wandzellen	Bode [29]
SK	skin cells	Sockel cells	Bode [29]
BM	Membrane cells	Wand cells	Bode [29]
BV1	Basivalvulae 1	Basivalvulae 1	Snodgrass [35],[36]
BV2	Basivalvulae 2	Basivalvulae 2	Snodgrass [35]
KZ	Channel cells	K+ anal Zellen	Bode [29]
DZ	Glandular cells	Drusen zellen	Bode [29]
Ag	ADR gland opening		Bode [29]
EP	Epiproct		Snodgrass [35]
PP	Paraproct		Snodgrass [35]
Ovipositor shaft	Anatomical cluster composed of 1st and 2nd valvulae		Snodgrass [35],[36]

Table S2. Muscles annotation and terminologies proposed.

Muscle name	Muscle type	Tergite	Muscle originate	Muscle insert	Equivalent muscle names			
					Snodgrass [35]	Davies [34]	Bode [29]	Zhou and Rédei [37]
**Muscle spermathecal basivalvula**	Unbranched	VII	Dorsal to the spermatheca	Inserted into the basi-valvulae of the ovipositor shaft				
**Inter tergite muscle 8**	Unbranched	VIII	VII	VIII		i.t.vlv8 Inter tergite muscle 8	1b	
**outer tergal valvilis 8**	Unbranched	VIII	antero lateral corner of 8th tergum	Valvilis		outer tergal valvilis 8 (o.t.vlv 8)	1a	
**Muscle ovary oviducts ADR gland**	Mooag		Ovary	oviduct, spermatheca				
**Muscle latero tergo postero Appendage gland 7 (ltpagm7b1)**	Branched	VII	Tergum	ADR gland				
	lptagmb1	VII	Tergum	ADR gland				
	lptagmb2	VII	Tergum	ADR gland				
	lptagmb3	VII	Tergum	ADR gland				
	lptagmb4	VII	Tergum	ADR gland				
	lptagmb5	VII	Tergum	ADR gland				

**Tergo spermatheca muscle**	Branched	VII	Tergum	Spermatheca				
	tsm1	VII	Tergum	SM2 and SM3				
	tsm2	VII	Tergum	SM3				
	Atsmps	VII	Spermatheca	SM2				
**Spermatheca basivalvulae muscle**	Unbranched Sbm	VII	Spermatheca	Basivalvula				
**Muscle spermatheca and vagina**	Msv	VII	Spermatheca	ADR gland & Vagina				
**Muscle tergo- valvilis posterior 9**	Branched (ovm1, ovm2, ovm3 & ovm4)	IX	Tergum	Valvula ovipositor		p.dv9	10	mtvp9
	ovm1	IX	Tergum	2nd valvifier				
	ovm2	IX	Tergum	2nd valvifier and attached laterally to the OVM1				
	ovm3	IX	Posteriorly attached to the Tergum 10	Attached with OVM2 and OVM4				
	ovm4	IX	Posteriorly attached to the	Attached with OVM3 and valvulae laterally and				

			Tergum 10	inserted into the 2nd valvifier				
	ovm5		Posteriorly attached to the Tergum 10	Attached with OVM3 and valvulae laterally and inserted into the 2nd valvifier				
**Muscle tergo-valvilis primus 8**	Mtv	VIII	Tergum	Apodeme plate 8				Muscle tergo-valvilis primus 8mtv8
**Tergo valvilis medial 10**	Unbranched	X	Tergum	2nd valvula tip		Transverse tergosternal ts10	16	Tergo valvilis medial 10
**Muscle longitudinal inter-tergite X**	Unbranched	X	Transverse tergite 11	Tergite 9		Muscle longitudinal inter-tergite X	15	
